# Cradle-to-gate life cycle assessment of sisal/PLA biocomposite: Uncovering the dominance of transport and burden-shifting trade-offs in sustainable material design

**DOI:** 10.1371/journal.pone.0349715

**Published:** 2026-09-23

**Authors:** Nickson Severian Kahigi, Revocatus Machunda, Josephine Joseph Mkunda, Mwema Felix Mwema

**Affiliations:** 1 School of Materials, Energy, Water and Environmental Science (MEWES), The Nelson Mandela African Institution of Science and Technology (NM-AIST), Arusha, Tanzania; 2 Department of Business Management, The Mbeya University of Science and Technology (MUST), Mbeya, Tanzania; 3 School of Business Studies and Humanities (BuSH), The Nelson Mandela African Institution of Science and Technology (NM-AIST), Arusha, Tanzania; King Mongkut’s University of Technology North Bangkok, THAILAND

## Abstract

Natural fibre-reinforced biocomposites are increasingly recognised as promising alternatives for reducing the high carbon footprint of the construction sector; however, the environmental implications of transcontinental supply chains remain insufficiently understood. This study presents a cradle-to-gate life cycle assessment of a sisal yarn-reinforced polylactic acid biocomposite plate manufactured in Denmark using sisal fibres cultivated and spun in Tanzania. Environmental impacts were assessed for a performance-based functional unit using the ReCiPe 2016 Midpoint (H) method, normalising values through a structural bending stiffness index to enable benchmarking against conventional window frame materials. Under baseline pilot-scale conditions, the biocomposite exhibited a global warming potential of 17.52 kg of carbon dioxide equivalent, with intercontinental air freight accounting for 62.53% of total emissions. Scenario optimisation involving ocean freight, 100% wind energy, and a 60:40 polylactic acid-to-sisal ratio reduced the global warming potential to 9.82 kg of carbon dioxide equivalent, a 43.97% reduction. When normalised for structural performance, the optimised biocomposite demonstrated a lower global warming potential than conventional polyvinyl chloride and aluminium window frame materials. Nevertheless, these improvements resulted in significant shifts in environmental burdens, with human non-carcinogenic toxicity increasing by 125.91% and freshwater ecotoxicity by 322.88%, primarily due to heavy fuel oil combustion during marine transport and the use of synthetic phosphate fertiliser in sisal cultivation. The findings indicate that although transcontinental sisal/polylactic acid biocomposites can offer competitive structural and climate performance, their long-term sustainability depends on holistic multi-indicator assessments and the adoption of cleaner logistics and sustainable agricultural practices to avoid transferring environmental burdens across impact categories.

## Introduction

The global building and construction sector is a primary driver of environmental degradation, accounting for approximately 37% of energy-related carbon dioxide emissions and nearly 34% of global energy demand [1]. In addition, the sector generates nearly 30% of global solid waste [2], consumes over 15% of global water resources [3], and accounts for approximately 50% of global raw material extraction [4]. Structural and semi-structural building components, such as window frames, cladding, and wall panels, have historically relied on primary timber resources for their renewability and favourable mechanical properties [5]. However, the Food and Agriculture Organisation projects that global roundwood demand could rise from 3.9 billion cubic metres annually to over 5.1 billion cubic metres by 2050, putting immense pressure on forest ecosystems and escalating the risks of deforestation, biodiversity loss, and habitat degradation [6–9].

To mitigate these ecological pressures, polymer composites have emerged as viable alternatives to conventional timber and synthetic structural elements [10]. However, conventional composites rely heavily on petroleum-based polymers and energy-intensive synthetic reinforcements such as glass and carbon fibres, generating significant greenhouse gas emissions and posing severe end-of-life disposal challenges [11,12].

Consequently, natural fibre-reinforced biocomposites, which combine lignocellulosic fibres with bio-based, biodegradable polymers such as polylactic acid (PLA), represent a highly promising paradigm shift [13,14]. Among natural fibres, sisal (Agave sisalana) has attracted attention for its rapid growth cycle, low agrochemical requirements, resilience to water stress, and high tensile strength [15]. While East African countries, particularly Tanzania, are major producers of high-grade sisal fibre, a substantial proportion is exported in raw form, limiting domestic value addition [9,16].

Recent research in biocomposite design has focused on innovative manufacturing routes and material blending strategies to overcome mechanical limitations and structural instabilities. For instance, blending high-density polyethene (HDPE) with poly (L-lactic acid) (PLLA) has been shown to reduce raw material costs and enhance structural integrity, particularly when processing temperature-sensitive matrices [17]. Additionally, composite materials incorporating continuous sisal fibre-reinforced PLA systems have been reported to improve mechanical, thermal, and rheological properties by optimising fibre configuration, material formulation, and manufacturing processes [18,19]. While these investigations demonstrate the technical feasibility of sisal/PLA composites, comparatively little attention has been given to quantifying their environmental performance using life cycle assessment, particularly under realistic production conditions.

Furthermore, additive manufacturing techniques, particularly 3D printing, have revolutionised the structural tailoring of these bio-based materials. Traditional 3D-printed biocomposites often suffer from fibre misalignment and poor bonding due to the use of short or randomly oriented fibres [20]. By contrast, novel techniques such as strategically interleaving continuous natural fibre cores within a PLA matrix have yielded dramatic mechanical improvements [18]. Specifically, strategically interleaved continuous sisal fibre cores treated with sodium hydroxide (NaOH) have been shown to increase tensile strength by 36.19% and interlaminar shear strength by 46.25% compared with neat PLA, achieving a tensile strength of 40.87 MPa and a tensile modulus of 2.42 GPa, alongside superior impact energy absorption and viscoelastic damping performance [18].

Despite these physical and mechanical advances, a material’s bio-based origin does not inherently ensure overall environmental sustainability across its production chain [21]. Yet, existing life cycle assessment studies of natural fibre composites focus predominantly on highly localised, regionally integrated European, North American, or Asian supply chains [22,23]. In this regard, there remains a significant gap in the literature on the environmental performance of biocomposites with geographically dispersed, transcontinental supply chains, especially those that obtain agricultural raw materials from developing African economies and export them to industrialised nations for advanced composite manufacturing [24].

Nevertheless, challenges associated with fibre–matrix compatibility, fibre hydrophilicity, and interfacial adhesion remain significant barriers to the industrial adoption of emerging biocomposites [25,26]. Consequently, environmental sustainability of these materials should be evaluated alongside processing technology and structural performance, making life cycle assessment an essential decision-support tool for the development of next-generation biocomposites [27,28].

This study contributes to addressing these gaps by presenting a comprehensive cradle-to-gate life cycle assessment of a sisal yarn-reinforced PLA composite plate manufactured in Denmark using sisal fibres cultivated, decorticated, and spun into yarns in Tanzania. By applying the ReCiPe 2016 Midpoint (H) method, this study quantifies midpoint environmental impacts, maps process-level hotspots, and explicitly examines the multi-indicator environmental trade-offs (burden shifting) that occur during supply-chain optimisation, logistics substitution, and polymer composition modification [29].

## Materials and methods

### Goal, scope, and system boundaries

This study applies a cradle-to-gate life cycle assessment to evaluate the environmental performance of a sisal yarn-reinforced PLA composite plate intended for potential construction applications, such as window panels and lightweight structural beams. Although the present study adopts a cradle-to-gate system boundary, this approach is consistent with evaluating emerging technologies during early development stages, when reliable information on service life, maintenance, reuse, recycling, and end-of-life management is unavailable [30]. ISO 14044 recommends that system boundaries be defined according to the study goal and available data while clearly acknowledging associated limitations [31].

Consequently, the present assessment focuses on identifying production-stage environmental hotspots and optimisation opportunities, whereas future cradle-to-grave assessments should incorporate experimentally validated durability, maintenance, and end-of-life scenarios once commercial deployment becomes feasible.

**Fig 1:**
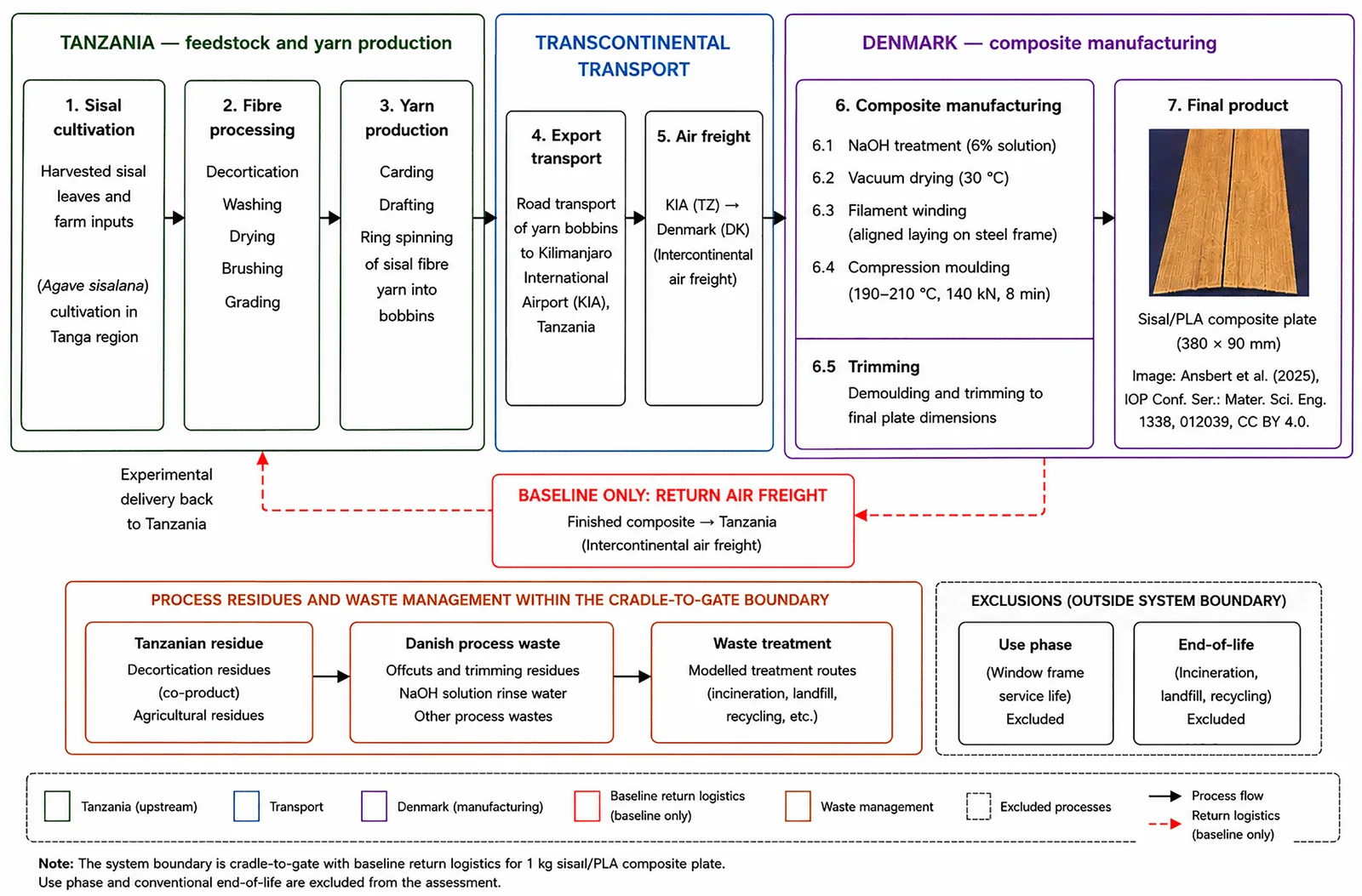
Cradle-to-gate system boundary of transcontinental sisal/PLA composite production. The diagram shows sisal cultivation and fibre/yarn production in Tanzania, domestic transport, air freight to Denmark, composite manufacturing (6% sodium hydroxide treatment, vacuum drying, filament winding, compression moulding and trimming), process-residue treatment, and the baseline return air transport of the finished composite to Tanzania. The final composite photograph is reproduced from Ansbert et al. [32] under the Creative Commons Attribution 4.0 (CC BY 4.0) licence.

The system boundary includes raw material production, sisal fibre processing, yarn production, domestic and intercontinental transportation, composite manufacturing, and waste management of process residues. The baseline also includes return air transport of the finished composite to Tanzania because this leg formed part of the experimental R&D supply chain. The use phase and conventional end-of-life of the finished composite are excluded. The system boundary is illustrated in Fig 1, highlighting the material and energy flows from sisal cultivation and yarn production in Tanzania through transport and composite manufacturing in Denmark.

### Functional unit and structural performance-based design equivalence

The primary functional unit of this study is defined as 1 kg of finished sisal/PLA composite plate, enabling direct comparison with previous life cycle assessment studies on natural fibre composites, in which mass-based functional units are commonly adopted for prototype materials [23]. While this approach facilitates benchmarking, it does not adequately capture the structural performance requirements of construction applications, such as window frames that must withstand bending stresses induced by wind loads [30]. To overcome this limitation, a performance-based functional unit was applied.

For a window-frame member subjected to bending under wind loading, the principal structural objective is to minimise mass while satisfying a prescribed flexural stiffness requirement. Following Ashby's materials selection methodology, the mass (m) of a structural member of length (L) under stiffness-controlled bending is inversely proportional to the material performance index. For members with constrained geometric optimisation, the appropriate material index is expressed as:


$$\begin{array}{c} M=\frac{\sqrt[{\text{3}}]{E}}{\rho } \end{array}$$ (1)

where (E) is the Young’s modulus (GPa), representing material stiffness, and (ρ) is the material density [33]. Materials with larger values of (M) provide greater stiffness per unit mass and are therefore preferred for lightweight bending-stiff structures [34]. Accordingly, for materials designed to provide equivalent structural performance, the required mass is inversely proportional to material index and can be expressed as:


$$\begin{array}{c} m=L^{\text{2}}\sqrt{S\times L}\left(\frac{\rho }{\sqrt[{\text{3}}]{E}}\right) \end{array}$$ (2)

By incorporating this performance-based functional unit, the environmental impacts of alternative materials can be normalised according to equivalent structural functionality rather than mass alone, enabling a more meaningful comparison for construction applications. In this study, the reference sisal/PLA composite has a density of 1.24 g/cm³ and a tensile modulus of 2.42 GPa [18,35]. Based on these properties, the relative mass required to achieve equivalent bending stiffness was evaluated and compared with conventional window frame materials, including timber, polyvinyl chloride (PVC), and structural aluminium [35].

### Life cycle inventory and composite manufacturing

#### Raw material extraction and processing logistics.

Primary foreground inventory data were collected on-site from commercial plantations and processing mills in the Tanga region of Tanzania, and paired with manufacturing laboratory data from the Risø Campus of the Technical University of Denmark (DTU) [32]. For the Tanzanian feedstock phase, sisal leaves were harvested from mature plants at their tenth harvest. The leaves were mechanically decorticated to maximise fibre recovery efficiency and reduce chemical dependency [36,37]. The resulting technical fibres were washed to remove chlorophyll and impurities, sun-dried, brushed, and graded. The 3L (3 meters long) grade was selected for its suitability as structural composite reinforcement [32]. Cultivation and extraction inventories include synthetic nitrogen and phosphate fertilisers applied at the nursery stage, diesel for agricultural machinery, grid electricity, and process water for leaf decortication [38–40].

Following extraction, the fibres were transported to a domestic spinning factory. Primary operational data, including electrical energy and auxiliary lubricant consumption during carding, drafting, and ring spinning, were directly measured by machine operators. Ring spinning was specifically utilised to optimise yarn uniformity and fibre cohesion [37], with the finished yarn wound onto bobbins for transcontinental transport.

#### Composite plate fabrication and conditioning.

The matrix phase consisted of primary poly(lactic acid) (PLA) filaments supplied on bobbins by Comfil ApS (Denmark), featuring a baseline density of 1.24 g/cm^3^ [32]. Composite plates were fabricated at RiSo DTU using a hybrid filament-winding–compression-moulding process to ensure controlled directional reinforcement and structural consistency [41].

To improve interfacial fibre–matrix adhesion, a subset of the sisal yarn was subjected to an alkali treatment using a 6% sodium hydroxide (NaOH) solution for 24 hours, followed by rinsing and oven-drying, conditioned at 50°C for 8 hours to remove hemicellulose, waxes, and surface impurities [42]. Untreated yarn was preserved as a baseline reference scenario for subsequent sensitivity analysis.

For plate assembly, a rectangular steel frame (490 mm × 240 mm × 2 mm) was mounted on a semi-automated filament-winding machine. The sisal yarn and PLA filaments were wound in a symmetric, layered configuration (inner PLA, middle sisal, outer PLA) under a controlled winding tension of 2–6 N to prevent yarn rupture. Prior to thermal consolidation, the wound assemblies were dried in a vacuum dryer estimated at 150 W for 24 hours to eliminate residual moisture and mitigate void formation.

The dried assemblies were subsequently consolidated via compression moulding at approximately 5 kW and a heating temperature set at 190°C–210°C for 8 minutes under a vacuum of approximately −700 mbar to facilitate optimal matrix melting and fibre impregnation. The heated laminate was then pressed at 140 kN (≈3 MPa) for 2 minutes at 30°C, followed by natural cooling, demoulding, and trimming to yield final plates measuring approximately 380 mm × 90 mm × 3.5 mm.

#### Foreground data inventory and energy modelling.

Material and energy flows for chemical treatment, filament winding, vacuum drying, compression moulding, and trimming were matched against experimental procedures adapted from Ansbert et al. [32]. The mass of a single trimmed composite plate was calculated to be 0.158 kg, based on its physical dimensions and a composite density verified by the rule of mixtures. Consequently, mass balancing was employed to scale all inputs and outputs to a reference functional unit (FU) of 1 kg of finished composite plate (≈6.33 plates/FU).

Because primary electricity demand was not directly metered during individual laboratory operations, an engineering calculation approach was applied. The electrical energy consumption ($E_{i}$) for each unit operation was determined using the following formula:


$$\begin{array}{c} E_{i}=P_{i}\times t_{i} \end{array}$$ (3)

Where: $E_{i}$ is the electricity consumption (kWh) for equipment component i, $P_{i}$ is the average electrical power rating of the specific equipment (kW), and $t_{i}$ is the total active operating time (h).

The electricity consumption for composite fabrication was estimated using each processing unit’s rated power and operating time. The WSH-1–4-2M-Flex filament-winding system was assumed to consume 1.5 kW over 25 min, followed by vacuum drying in a laboratory oven rated at 0.5 kW for 50 min. Compression moulding was performed using a 5 kW heat press for 8 min, while post-winding vacuum drying was performed using a 150 W vacuum pump for 24 h. A 3 kW hydraulic heat press was subsequently used to consolidate the composite for 8 min. All the processing parameters and operating durations were reported in Ansbert et al. [32]. The allocation for electricity use in finalisation stages, including cooling, manual handling, testing, weighing, and trimming operations, was excluded from the energy inventory because their power consumption was assumed to be negligible for the functional unit considered.

#### Background data, modelling, and data quality.

Life Cycle Inventory modelling was performed using SimaPro version 10.2.0.2 software. Secondary background data on electricity grids, synthetic fertiliser production, polylactic acid (PLA) resin synthesis, transport services, and municipal waste treatment were obtained from the ecoinvent database v3.11 using the cut-off allocation model. All selected background datasets were verified to ensure they either directly represent “global data” (GLO) or serve as representative technological and geographical proxies for the “Rest-of-the-World” (RoW) region. An inventory data quality assessment was conducted using the ecoinvent pedigree matrix methodology [43], based on the quality indicators: reliability, completeness, temporal correlation, geographical correlation, and technological correlation.

Primary foreground inventory data were collected through field observations, direct operational measurements, laboratory manufacturing records, and interviews with technical personnel involved in sisal cultivation, fibre processing, yarn production, and composite fabrication. Engineering calculations were verified against equipment specifications and experimental operating schedules before being integrated into the life cycle inventory.

Data quality was then assessed using the ecoinvent pedigree matrix, with each indicator assigned a score from 1 (very good) to 5 (very poor) based on reliability, completeness, and temporal, geographical, and technological representativeness. Primary data from sisal cultivation and yarn production in Tanzania, laboratory composite manufacturing in Denmark, and ecoinvent background datasets received high-quality scores, whereas engineering estimates of energy consumption for the Danish manufacturing process were assigned lower scores due to greater uncertainty. The pedigree assessment presented in Table 1, provides a transparent evaluation of inventory quality and supports the interpretation of uncertainty in the life cycle inventory.

**Table 1:** Pedigree matrix assessment of the life cycle inventory.

Inventory dataset	Data source	Reliability	Completeness	Temporal correlation	Geographical correlation	Technological correlation
Sisal cultivation	Primary field data	1	1	1	1	1
Fibre extraction and drying	Primary measurements	1	1	1	1	1
Sisal yarn production	Primary industrial data	1	1	1	1	1
PLA production	ecoinvent v3.11	1	1	1	1	1
Electricity (Denmark)	ecoinvent v3.11	1	1	1	1	1
Filament winding electricity	Engineering estimate	3	3	2	2	2
Vacuum drying electricity	Engineering estimate	3	3	2	2	2
Compression moulding electricity	Engineering estimate	3	3	2	2	2
Road transport	ecoinvent v3.11	1	1	1	1	1
Air freight	ecoinvent v3.11	1	1	1	1	1
Sea freight (scenario analysis)	ecoinvent v3.11	1	1	1	1	1

Scoring criteria: 1 = very good; 2 = good; 3 = fair; 4 = poor; 5 = very poor.

### Life cycle inventory analysis

Building upon the data sources, modelling parameters, and allocation rules described earlier, the consolidated life cycle inventory (LCI) data required to produce 1 kg of finished sisal-reinforced PLA composite plate were generated and organised in Table 2. All foreground and background processes within the defined cradle-to-gate system boundary, related to resource extraction, handling, material processing, and intercontinental shipment, are explicitly quantified and presented.

**Table 2:** Consolidated life cycle inventory data for 1 kg of sisal-reinforced PLA composite plate.

Production Phase	Flow	Unit	Quantity	Data Source / Quality
Sisal Cultivation (TZ)	Dry sisal fibre	kg	0.30	Primary data (Tanga, Tanzania)
	NPK fertilizer	kg	0.0047	Primary data / ecoinvent v3.11
	Diesel	L	0.056	Plantation logs / ecoinvent v3.11
	Electricity (TZ grid)	kWh	0.060	Tanzanian electric utility records
	Process water	${\text{m}}^{\text{3}}$	0.195	Site report
	Decortication residue	kg	0.58	Mass balanced co-product
Yarn Production (TZ)	Sisal yarn	kg	0.28	Carding & spinning logs
	Electricity	kWh	0.42	Metered spinning mill consumption
	Lubricants	kg	0.002	Auxiliary inputs
Domestic Transport (TZ)	Farm → Decortication	t·km	0.100	Lorry, fleet average (ecoinvent)
	Decortication → Spinning	t·km	0.0255	Lorry, fleet average (ecoinvent)
	Spinning → Export Point	t·km	0.140	Lorry, fleet average (ecoinvent)
Export Logistics	Air freight (TZ → DK; yarn)	t·km	1.96	Cargo aircraft payload factor for 0.28 kg of yarn.
Local Road Transport (DK)	Local Road Transport (DK)	t·km	0.02	Lorry, 16–32 t (ecoinvent)
Composite Mfg. (DK)	PLA resin filament	kg	0.72	Primary data (Comfil ApS), reported in Ansbert et al. [32]
	NaOH	kg	0.010	Chemical treatment log
	Electricity (DK grid)	kWh	5.34	Compression moulding/winding
	Solvents	kg	0.06	Process cleaning
	Composite offcuts	kg	0.05	Trimming waste
Composite delivery (DK→TZ)	Shipping of trimmed composite (Air)	t.km	7.0	1 kg of finished composite delivered to Tanzania to finalise the experimental route.
	Road (KIA → NM-AIST)	t·km	0.04	Light commercial vehicle

TZ = Tanzania; DK = Denmark; PLA = Polylactic acid; NaOH = Sodium hydroxide; NPK = Nitrogen-Phosphorus-Potassium; KIA = Kilimanjaro International Airport; NM-AIST = Nelson Mandela African Institution of Science and Technology; t·km = tonne-kilometre; kWh = kilowatt-hour.

### Access to primary data

Primary life cycle inventory (LCI) data were obtained through authorised field and laboratory access in collaboration with the Nelson Mandela African Institution of Science and Technology (NM-AIST), the Tanzania Sisal Board (TSB), and project partners. No additional governmental research permit or ethical approval was required because the study involved industrial process data collection and observations at commercial production facilities. Access to the Tanzanian field sites was authorised through the Tanzania Sisal Board (TSB). Data for the sisal/PLA composite manufacturing process were obtained through collaboration with the researcher responsible for sisal/PLA prototype development at the Technical University of Denmark (DTU Risø Campus). The manufacturing methodology and experimental procedures were documented by Ansbert et al. [32].

### Life cycle impact assessment and allocation framework

Environmental impacts were modelled using SimaPro software (version 10.2.0.2) and characterised using the ReCiPe 2016 Midpoint (H) v1.1 / World (2010) H method. The ReCiPe framework was selected because it provides robust characterisation of climate change, toxicity, land use, and resource depletion indicators that are highly relevant to agricultural-based composite systems [32,44]. The evaluated midpoint impact categories included: Global Warming Potential (${\text{kg CO}}_{\text{2}}-\text{eq}$), Fossil Resource Scarcity Potential (kg oil−eq), Land Use (${\text{m}}^{\text{2}}\text{a crop}-\text{eq}$), Ionising Radiation Potential (kBq Co−60−eq), Terrestrial, Freshwater, and Marine Ecotoxicity Potentials (kg 1,4−DCB−eq), and Human Carcinogenic and Non-Carcinogenic Toxicity Potentials (kg 1,4−DCB−eq).

To maintain methodological consistency within the inventory, environmental burdens from the agricultural extraction phase were partitioned using a mass-allocation procedure. Mechanical wet-decortication of sisal leaves yields approximately 4% dry weight of technical fibres with 3 metres long (grade 3L) and 96% wet weight of residue, comprising organic waste and process water) [45,46]. Because the technical fibre is the primary economic driver of the sisal plantation, 100% of upstream cultivation and agricultural inputs were allocated to the extracted dry fibre. The organic residue was managed locally as process waste through open dumping into available infrastructure, often along the processing site.

### Scenario-based sensitivity and Monte Carlo uncertainty analysis

To validate the ecological hotspots identified in the baseline life cycle assessment and to evaluate the model’s structural robustness, a dual-investigation approach comprising scenario-based sensitivity analysis and statistical uncertainty analysis was implemented. Within this framework, three distinct configurations were modelled to assess the practical viability of alternative logistics, manufacturing energy sources, and raw-material formulations relative to the experimental baseline.

The first configuration, designated as Scenario O (Baseline), models the empirical pilot-scale parameters as documented during the actual project execution. This baseline encompasses intercontinental air freight to ship processed yarns from Tanzania to Denmark, the reciprocal return air transport of finalised composite samples back to Tanzania, the standard Danish grid electricity mix for manufacturing operations, and a 72:28 polymer-to-fibre mass ratio.

The second configuration, Scenario A (Moderate Optimisation), alters the baseline logistics by substituting high-impact intercontinental air freight with marine container shipping. To lower matrix dependency and its associated environmental burdens, the composite formulation is adjusted to a 60:40 polylactic acid (PLA)-to-sisal mass ratio. Additionally, standard Danish grid electricity is substituted with 100% wind-based renewable energy, and upstream agricultural chemical inputs and process water consumption at the Tanzanian cultivation phase are reduced by 10%.

The third configuration, Scenario B (Advanced Optimisation), evaluates the compounding effects of deeper manufacturing and agricultural interventions. This scenario incorporates a more aggressive 20% mass reduction in Tanzanian agricultural inputs, completely replaces the 6% NaOH chemical treatment in Denmark with a clean water-based thermal treatment process, and sets the material composition to a 65:35 PLA-to-sisal mass ratio. The definitive parameters and technical justifications governing these scenarios are presented in Table 3.

**Table 3:** Parametric modifications in sensitivity analysis scenarios.

Life-Cycle Stage	Baseline Condition (Scenario O)	Scenario A (Moderate Optimisation)	Scenario B (Advanced Optimisation)	Technical Justification
Intercontinental Shipping	Air Freight	Sea Freight (Container)	Sea Freight (Container)	Shifting from cargo aircraft (500−1,000 g/t·km) to container vessels (10−40 g/t·km).
Grid Electricity (DK)	Conventional Danish Grid	100% Wind Power	100% Wind Power	Decarbonising energy-intensive processing (compression moulding, drying).
PLA: Sisal Mass Ratio	72:28	60:40	65:35	Lowering starch-crop polymer dependency to reduce land and water use.
Chemical Treatment	6% NaOH Alkali Wash	3% NaOH Alkali Wash	Water-Based Thermal	Reducing chemical synthesis demands and ecotoxic waste streams.
Agrochemical Inputs (TZ)	Baseline Cultivation	10% Mass Reduction	20% Mass Reduction	Minimising nitrogen and phosphate fertiliser runoff in soil and water.

Scenario A represents moderate optimisation, while Scenario B represents advanced optimisation. Changes involve lower-emission intercontinental transport, renewable electricity, increased sisal content, reduced chemical intensity, and lower agrochemical inputs.

Moreover, to assess the reliability and internal consistency of the baseline life cycle impact assessment (LCIA) outputs, a stochastic uncertainty analysis was performed using Monte Carlo simulation, implemented in MS Excel. The simulation was executed over 10,000 iterations to assess the statistical stability of the primary impact categories and to calculate 95% confidence intervals (CIs) for the deterministic estimates.

Although air freight is not the preferred logistics option for commercial production, especially for return logistics, it accurately reflects the mode of transportation used during the experimental development of the present prototype. Therefore, in the baseline scenario, air freight service represents the actual research and development (R&D) supply chain rather than a hypothetical industrial system. Subsequently, the marine freight system considered in the improvement scenarios highlights potential commercial logistics by replacing air transport with marine freight, distinguishing between prototype-scale environmental performance and expected industrial deployment and providing a realistic pathway for future commercialisation along with optimised logistics configurations [24].

## Results

### Baseline environmental impact characterisation

The baseline environmental performance of the sisal-reinforced PLA composite was characterised using SimaPro version 10.2.0.2 with the ReCiPe 2016 Midpoint (H) v1.1 method. Proportional process-level contributions for the principal midpoint categories are detailed in Table 4.

**Table 4:** Baseline midpoint environmental impacts and process contributions (per 1 kg of sisal/PLA composite).

Impact Category	Unit	Composite Mfg. (DK)	Intercontinental Air Freight	Local Transport (TZ & DK)	Sisal Fibre (TZ)	Sisal Yarn (TZ)	Waste Treatment (DK)	Total
GWP	kg CO_2_-eq	3.477 (19.84%)	10.957 (62.53%)	0.297 (1.70%)	1.700 (9.70%)	0.792 (4.52%)	0.300 (1.71%)	17.523
IRP	kBq Co-60-eq	0.426 (65.94%)	0.095 (14.71%)	0.003 (0.46%)	0.092 (14.24%)	0.028 (4.33%)	0.003 (0.46%)	0.646
OFHH	kg NOx-eq	0.008 (11.94%)	0.051 (76.12%)	0.003 (4.48%)	0.003 (4.48%)	0.001 (1.49%)	0.000 (0.00%)	0.067
TAP	kg SO_2_-eq	0.012 (25.00%)	0.029 (60.42%)	0.001 (2.08%)	0.004 (8.33%)	0.002 (4.17%)	0.000 (0.00%)	0.048
FEP	kg P-eq	0.003 (16.67%)	0.001 (5.56%)	0.000 (0.00%)	0.011 (61.11%)	0.003 (16.67%)	0.000 (0.00%)	0.018
TETP	kg 1,4-DCB	7.883 (45.71%)	5.172 (29.99%)	1.116 (6.47%)	1.788 (10.37%)	0.959 (5.56%)	0.327 (1.90%)	17.245
HCTP	kg 1,4-DCB	0.132 (33.33%)	0.058 (14.65%)	0.002 (0.51%)	0.115 (29.04%)	0.035 (8.84%)	0.054 (13.64%)	0.396
HNCTP	kg 1,4-DCB	2.283 (38.58%)	1.125 (19.01%)	0.035 (0.59%)	1.650 (27.89%)	0.513 (8.67%)	0.310 (5.24%)	5.917
LU	m^2^a crop-eq	1.693 (90.20%)	0.153 (8.15%)	0.004 (0.21%)	0.017 (0.91%)	0.009 (0.48%)	0.001 (0.05%)	1.877
FRSP	kg oil-eq	0.946 (18.38%)	3.499 (67.98%)	0.095 (1.85%)	0.374 (7.27%)	0.200 (3.89%)	0.032 (0.62%)	5.147
WCP	m^3^	0.146 (86.90%)	0.001 (0.60%)	0.000 (0.00%)	0.018 (10.71%)	0.011 (6.55%)	−0.009 (−5.36%)	0.168

GWP = Global Warming Potential; IRP = Ionising Radiation Potential; OFHH = Ozone Formation Potential, Human Health; TAP = Terrestrial Acidification Potential; FEP = Freshwater Eutrophication Potential; TETP = Terrestrial Ecotoxicity Potential; HCTP = Human Carcinogenic Toxicity Potential; HNCTP = Human Non-Carcinogenic Toxicity Potential; LU = Land Use; FRSP = Fossil Resource Scarcity Potential; WCP = Water Consumption Potential.

As indicated in Table 4, the environmental profile of the sisal/PLA composite is dominated by a limited number of impact categories, demonstrating that the overall environmental burden is driven by specific processes rather than being uniformly distributed across all indicators. Among the eighteen midpoint categories assessed, the most environmentally relevant impacts based on total characterised values include global warming potential (17.52 kg CO_2_-eq), terrestrial ecotoxicity potential (17.25 kg 1,4-DCB), human non-carcinogenic toxicity potential (5.92 kg 1,4-DCB), fossil resource scarcity potential (5.15 kg oil-eq), land use (1.88 crop-eq), and water consumption potential (0.168 m^3^).

**Fig 2:**
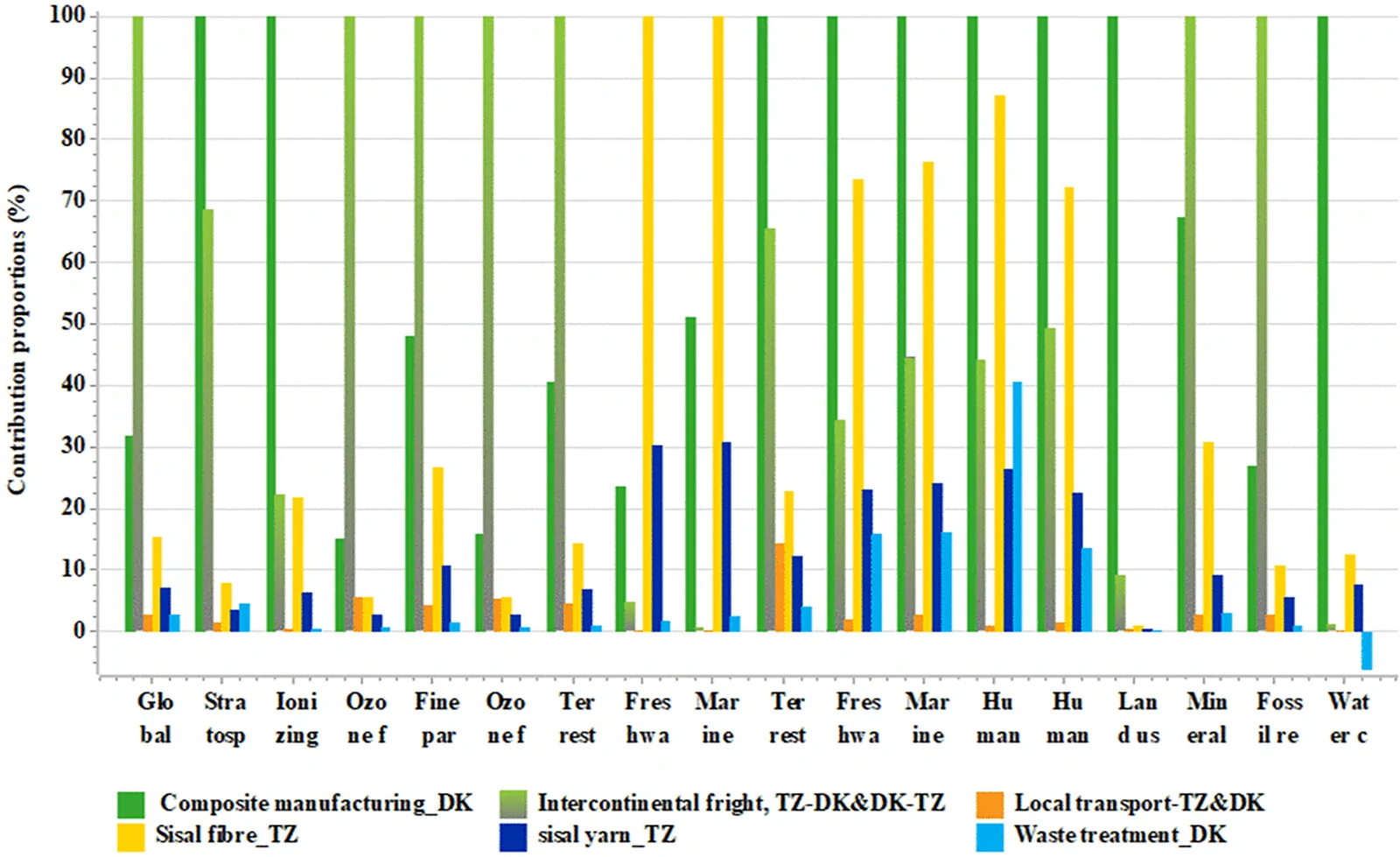
Process-level contributions to selected midpoint impacts of sisal/PLA composite production. The chart shows percentage contributions from composite manufacturing, intercontinental air freight, local transport, sisal fibre production, sisal yarn production, and waste treatment.

Climate change emerges as one of the most critical impact categories, with a total burden of 17.52 kg -eq per kilogram of composite. This is mainly associated with intercontinental air freight between Tanzania and Denmark, the dominant contributor, accounting for approximately 10.96 kg-eq, corresponding to almost 62.53% of total greenhouse gas emissions, as highlighted in Fig 2. This contribution is substantially higher than those associated with composite manufacturing in Denmark (19.84%), sisal fibre production in Tanzania (9.70%), and sisal yarn production (4.52%).

A similar dominance of air transport is observed across other combustion-related impact categories, including ozone formation (76.12% contribution to OFHH), terrestrial acidification (60.42% contribution to TAP), and fossil resource scarcity (67.98% contribution to FRSP). These results highlight the significant influence of fuel-intensive long-distance transportation, which contributes to emissions of nitrogen oxides, sulfur compounds, and particulate matter precursors, as well as increased fossil fuel depletion. Such findings are consistent with previous studies on bio-based materials with geographically dispersed supply chains, where transportation has been identified as a major environmental hotspot.

Composite manufacturing in Denmark is the second most significant contributor across several impact categories. This stage accounts for 65.94% of ionising radiation potential, 45.71% of terrestrial ecotoxicity, 33.33% of human carcinogenic toxicity, 90.20% of land use, and 86.90% of total water consumption. These impacts are largely driven by electricity use in vacuum drying, filament winding, and compression moulding, as well as upstream burdens from PLA resin production and chemical treatment inputs. The high land-use contribution aligns with the agricultural origin of PLA feedstocks, which typically rely on starch- or sugar-based crops. At the same time, elevated water consumption reflects both direct process water requirements and indirect water use embedded in polymer production datasets.

Sisal fibre production in Tanzania is another important hotspot, particularly in the nutrient enrichment and toxicity-related categories. This stage accounts for a substantial share of freshwater eutrophication (61.11% of FEP) and marine eutrophication, as well as human non-carcinogenic toxicity (27.89% of HNCTP). These impacts stem from the use of synthetic fertilisers during cultivation, water-intensive fibre washing during decortication, and diesel emissions from agricultural operations. This pattern reflects a common trade-off observed in natural fibre systems, where substituting synthetic reinforcement materials can increase environmental burdens at the agricultural and biomass-processing stages.

In contrast, sisal yarn production contributes relatively smaller shares across most impact categories. However, it remains relevant for selected indicators such as eutrophication (16.67% of FEP) and non-carcinogenic toxicity (8.67% of HNCTP). These contributions are primarily associated with electricity consumption and mechanical processing during spinning, suggesting that value-added processing stages introduce additional environmental burdens beyond fibre extraction. Local transportation within Tanzania and Denmark contributes only marginally to the overall impacts, generally accounting for less than 5% across most categories. Similarly, waste treatment processes have limited influence on the environmental profile, with minor contributions across most indicators and a small negative value for water consumption (−5.36%), reflecting avoided burdens within the waste management system.

### Sensitivity and scenario-based logistics and composition modifications

The comparative deterministic midpoint environmental impacts for the evaluated optimisation scenarios, along with their percentage variances relative to the baseline, are detailed in Table 5.

**Table 5:** Deterministic midpoint impacts analysis under sensitivity scenarios.

Impact Category	Unit	Scenario O (Baseline)	Scenario A (Moderate)	Scenario B (Advanced)	% Change (𝐎→𝐀)	% Change (𝐎→𝐁)
GWP	${\text{kg CO}}_{\text{2}}-\text{eq}$	17.523	9.818	9.695	−43.97%	−44.67%
TETP	kg 1,4−DCB	17.245	18.997	18.624	+10.16%	+8.00%
HNCTP	kg 1,4−DCB	5.917	13.367	13.413	+125.91%	+126.69%
FRSP	kg oil−eq	5.147	2.576	2.544	−49.95%	−50.57%
LU	${\text{m}}^{\text{2}}\text{a crop}-\text{eq}$	1.877	1.330	1.232	−29.14%	−34.36%
METP	kg 1,4−DCB	0.170	0.670	0.674	+294.12%	+296.47%
FETP	kg 1,4−DCB	0.118	0.499	0.502	+322.88%	+325.42%

Scenario O represents the baseline system, Scenario A represents moderate optimisation, and Scenario B represents advanced optimisation. Percentage changes are calculated relative to Scenario O; negative values indicate reductions in environmental impact, whereas positive values indicate increases.

The sensitivity analysis demonstrates that the environmental performance of the sisal/PLA composite is highly dependent on the transport mode, manufacturing electricity profile, and material composition. Shifting from air cargo to marine container transport in Scenario A drives down Global Warming Potential (GWP) by 43.97% (from 17.52 to 9.82 kg CO_2_-eq) and Fossil Resource Scarcity (FRSP) by 49.95% (from 5.15 to 2.58 kg oil−eq). This drastic reduction demonstrates that while intercontinental air freight was acceptable for pilot-scale R&D testing, it introduces prohibitive ecological burdens at scale. Shifting to commercial marine logistics represents the only viable path for stabilising the carbon profile of a transcontinental biocomposite supply chain.

Modifying the structural composition by lowering the matrix fraction from 72:28–60:40 (PLA-to-fibre ratio; Scenario A) reduces Land Use (LU) by 29.14% and FRSP by 49.95%. These findings reveal that, despite PLA's bio-based origins, its upstream agricultural feedstock cultivation and industrial polymerisation remain resource-intensive; maximising the reinforcing fibre volume fraction effectively decouples the composite from these polymer-associated hotspots.

Conversely, the advanced manufacturing adjustments and chemical-free thermal processes introduced in Scenario B yield diminishing marginal returns, reducing GWP by only an additional 1.30% relative to Scenario A. This indicates that once the primary logistics and processing energy vectors are fully decarbonised, the residual environmental burdens are rigidly governed by upstream background data constraints and agricultural parameters.

### Monte Carlo uncertainty analysis and statistical validation

Monte Carlo uncertainty analysis (MCA) comprising 10,000 iterations was implemented in Microsoft Excel. The supporting spreadsheet used normal probability distributions for the dominant foreground contribution processes, implemented with the Excel NORM.INV(RAND(), mean, standard deviation) function. Process-level coefficients of variation were 10% for composite manufacturing, 5% for intercontinental air freight, 10% for local transport, 20% for sisal fibre production, 15% for sisal yarn production, and 10% for waste treatment. The stochastic analysis covered five midpoint categories: GWP, TETP, HNCTP, LU, and FRSP, were selected for MCA, as these were considered key environmental burden based on their total magnitudes. The stochastic outputs from the 10,000-iteration Monte Carlo simulation are compiled in Table 6. The corresponding simulation data and calculations are provided as S1 File.

**Table 6:** Statistical summary of Monte Carlo uncertainty analysis (10,000 iterations).

Statistic	GWP (kg CO2-eq)	TETP (kg 1,4-DCB-eq)	HNCTP (kg 1,4-DCB-eq)	LU (m2a crop-eq)	FRSP (kg oil-eq)
Average	17.534	17.237	5.916	1.878	5.145
Standard deviation	0.748	0.943	0.414	0.170	0.213
CoV (%)	4.26	5.47	7.00	9.04	4.14
Lower 95% CI	16.055	15.392	5.110	1.543	4.723
Upper 95% CI	18.999	19.071	6.712	2.206	5.557
Median	17.530	17.231	5.915	1.878	5.145
Skewness	0.012	0.009	−0.010	−0.021	−0.036
Kurtosis	0.032	−0.084	0.020	−0.097	−0.011

**Fig 3:**
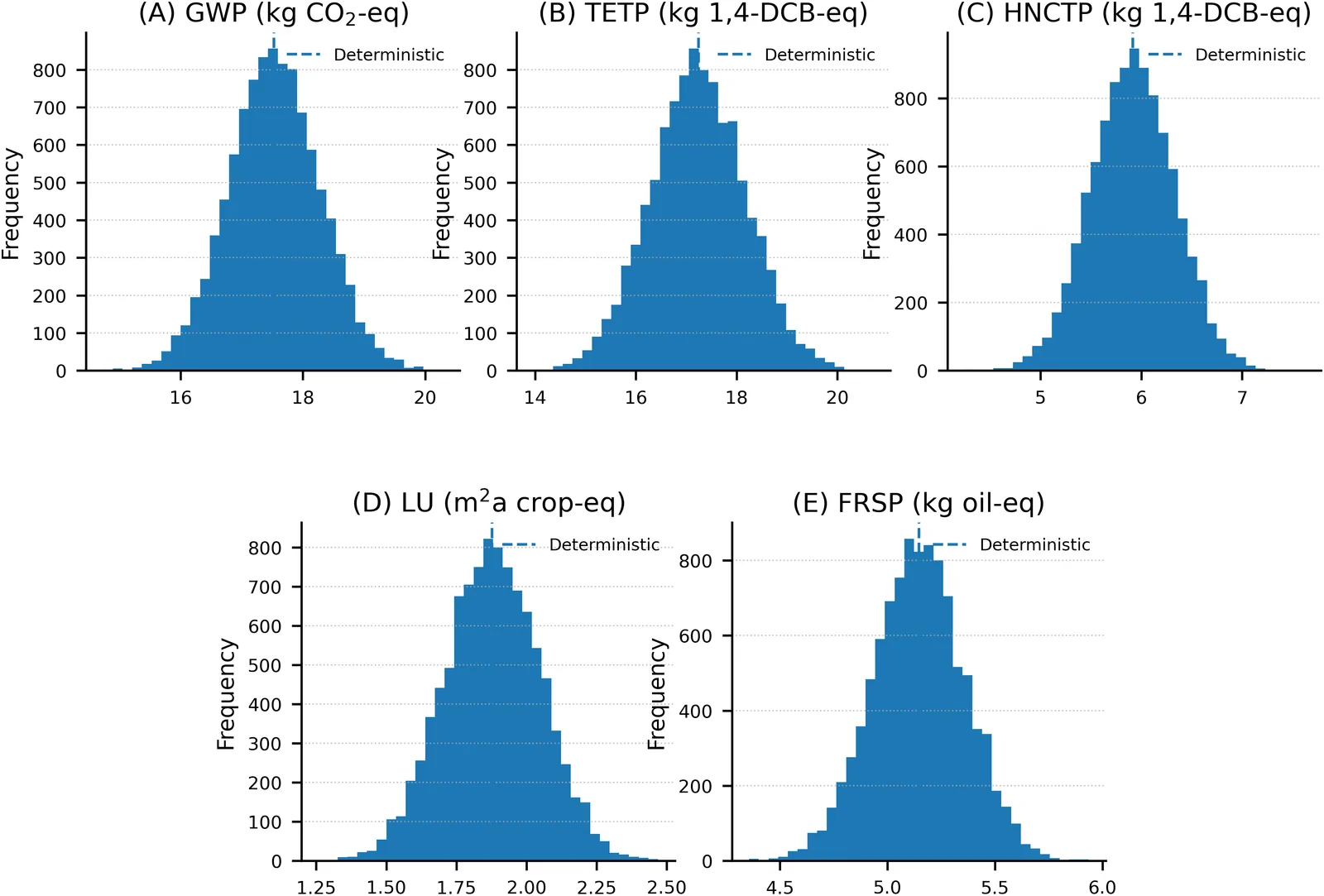
Monte Carlo uncertainty distributions for selected midpoint environmental impacts of sisal/PLA composite production. Panels show (A) global warming potential (GWP), (B) terrestrial ecotoxicity potential (TETP), (C) human non-carcinogenic toxicity potential (HNCTP), (D) land use (LU), and (E) fossil resource scarcity potential (FRSP) from 10,000 simulations. Dashed vertical lines indicate the deterministic baseline estimates.

GWP = global warming potential (kg CO_2_-eq); TETP = terrestrial ecotoxicity potential (kg 1,4-DCB-eq); HNCTP = human non-carcinogenic toxicity potential (kg 1,4-DCB-eq); LU = land use (m²a crop-eq); FRSP = fossil resource scarcity potential (kg oil-eq). The Monte Carlo simulation used the normal probability distributions implemented in S1 File. The resulting probability distributions for the selected impact categories are presented in Fig 3. The distributions are approximately symmetric, with simulated means closely matching the deterministic baseline estimates.

As shown in Table 6, the simulated means were very close to the deterministic baseline values, with absolute differences ranging from 0.006% for HNCTP to 0.065% for GWP. The 95% simulation intervals were 16.055–18.999 kg CO_2_-eq for GWP, 15.392–19.071 kg 1,4-DCB-eq for TETP, 5.110–6.712 kg 1,4-DCB-eq for HNCTP, 1.543–2.206 m²a crop-eq for LU, and 4.723–5.557 kg oil-eq for FRSP. The coefficients of variation ranged from 4.14% for FRSP to 9.04% for LU. The near-symmetric distributions and close agreement between deterministic and simulated means indicate that the principal environmental conclusions are not materially altered by the specified foreground uncertainty.

Overall, the Monte Carlo analysis supports the robustness of the identified environmental hotspots, particularly the dominance of intercontinental air freight for GWP and FRSP and the importance of composite manufacturing for toxicity-related impacts. The stochastic results therefore reinforce, rather than replace, the deterministic LCA interpretation.

## Discussion

### Comparison with prior natural fibre composite LCA studies

To contextualise the environmental performance of the sisal/PLA composite, its cradle-to-gate profile was first compared with existing life cycle assessment (LCA) literature on natural fibre composites. Previous research consistently indicates that natural fibre composites exhibit lower global warming potential (GWP) than conventional glass fibre or petroleum-based alternatives [47,48]. However, the distribution of environmental impacts across non-climate categories, such as ecotoxicity, land use, eutrophication, and human toxicity, varies considerably based on agricultural cultivation practices, transport logistics, matrix selection, and manufacturing processes.

In the present study, the baseline GWP was estimated at 17.52 kg CO_2_-eq per functional unit (1 kg of sisal/PLA composite), which is substantially higher than values reported for many European natural fibre systems. For instance, Seile et al. [23] reported GWP values of 1.19 kg CO_2_-eq/kg for flax/PLA and 1.70 kg CO_2_-eq/kg for hemp/PLA. These lower impacts were primarily attributed to regionally integrated supply chains within Europe and the absence of intercontinental air freight. Similarly, La Rosa et al.[49] demonstrated that flax-based automotive composites achieved 30–50% reductions in climate impacts compared to glass fibre counterparts, largely due to low-energy fibre processing.

The comparatively high baseline GWP observed in this study is predominantly driven by intercontinental air transportation between Tanzania and Denmark, which alone accounted for 62.6% (10.96 kg CO_2_-eq) of the total climate impact. In contrast, literature shows that transportation typically accounts for less than 10–20% of total GWP in geographically localised supply chains [23]. When air freight was replaced with marine transport in Scenario A, the GWP decreased by 43.9% to 9.82 kg CO_2_-eq. This substantial reduction underscores the critical role of logistics configuration in determining the ultimate viability of bio-composites.

Another distinguishing feature of this system is the elevated contribution of toxicity-related categories, specifically terrestrial ecotoxicity (17.25 kg 1,4-DCB-eq) and human non-carcinogenic toxicity (5.92 kg 1,4-DCB-eq). These are orders of magnitude higher than the burdens reported by Seile et al. [23], where terrestrial ecotoxicity for flax/PLA was approximately 0.005 kg 1,4-DCB-eq and human toxicity remained below 1 kg 1,4-DCB-eq. These elevated burdens are primarily associated with synthetic fertiliser application during the sisal nursery stage, sodium hydroxide treatments during processing, electricity consumption, and transport emissions. Similar hotspot trends have been identified by Korol et al. [48] and Kahigi et al. [50], who noted that agricultural chemical inputs dominate toxicity profiles via raw material production, pesticide application, and heavy metal releases.

In terms of material composition, the PLA matrix emerged as a significant environmental hotspot, contributing heavily to fossil resource scarcity (5.15 kg oil-eq) and land use (1.88 m^2^a-eq). These findings align with those of Madival et al. [51], who demonstrated that although biopolymers are renewable, their upstream cultivation and polymerisation remain energy- and resource-intensive. Optimising the current composite by reducing the PLA-to-sisal ratio from 72:28 to 60:40 resulted in a 29.1% reduction in land-use impacts, aligning with prior recommendations to lower polymer fractions to maximise eco-efficiency.

Despite these supply-chain hot spots, the sisal/PLA composite still demonstrates environmental advantages over conventional synthetic polymers. For example, Seile et al. [23] reported that PA66/glass fibre composites emit 9.14 kg CO_2_-eq/kg, which is almost similar to the emissions reported under scenario A and B in the present study. However, when compared to hemp or flax-based composites, sisal/PLA and PA66/glass composites are roughly 81–87% worse in terms of carbon emissions, but in terms of energy saving, these composites require about 60–80% less energy compared to glass fibre composites [52].

Conversely, the interventions explored in this study (e.g., renewable electricity, reduced polymer content, and maritime logistics) revealed an environmental trade-off that is frequently overlooked in the literature. While these strategies successfully reduced GWP and fossil scarcity, they induced a burden-shifting effect, inadvertently increasing toxicity-related impacts, aligning with findings by Kanchiralla et al. [53]. This underscores the risk of relying on carbon-centric metrics alone, which can overlook upstream toxicity trade-offs associated with energy systems, infrastructure, and material extraction.

### Performance-based structural-environmental benchmarking

Building on mass-based comparative LCA, which can be misleading, especially when comparable materials have different mechanical properties, a performance-based structural-environmental benchmarking framework that uses global warming potential (GWP) as a measure of climate change was employed to evaluate the composite’s environmental potential for construction applications. This approach combines cradle-to-gate GWP with material index (M) modelling for minimum weight under bending-stiffness constraints, ensuring that alternative window-frame materials are compared on an equivalent structural performance basis for identical wind-loading conditions.

Reference engineering parameters and environmental profiles for conventional window frames (structural timber, PVC, and structural aluminium) were synthesised from standard industry baselines. The present performance analysis of the sisal/PLA biocomposite used a density of ρ = 1.24 g/cm3 obtained from [35], and a Young’s modulus of E = 2.42 GPa from [18]. This was used to calculate the structural material index () of approximately 1.08 for the sisal/PLA composite, assuming a minimum-weight plate application, with limited stiffness and bending. This established the baseline from which equivalent structural masses and subsequent GWP impacts were calculated and compared with those of other materials, as presented in Table 7 [18,35,54].

**Table 7:** Performance-based environmental benchmarking against alternative window frame materials.

Window Frame Material	Density (ρ, g/cm^3^)	Young’s Modulus (E, GPa)	Material Index (M)	Equivalent Mass for Stiffness (kg)	Cradle-to-Gate GWP (kg CO_2_-eq/ kg)	Embodied GWP per Structural Frame (kg CO_2_-eq)
Timber Frame	0.50	10.0	4.31	10.00	1.30	13.0
PVC Frame	1.40	3.0	1.03	41.80	4.41	184.3
Aluminium Frame	2.70	70.0	1.53	28.20	17.23	485.9
Sisal/PLA (Scenario O)	1.24	2.42	1.08	39.90	17.52	699.0
Sisal/PLA (Scenario A)	1.24	2.42	1.08	39.90	9.82	391.8
Sisal/PLA (Scenario B)	1.24	2.42	1.08	39.90	9.70	387.0

GWP denotes global warming potential. Equivalent mass is the mass required to achieve the same stiffness as the reference structural frame based on the reported material index. Embodied GWP is calculated from the cradle-to-gate GWP per kilogram and the corresponding equivalent structural mass; Scenarios A and B represent the moderate and advanced optimisation configurations, respectively.

Performance-based benchmarking provides critical insights into material selection for structural designs. On a simple mass basis (1 kg of material), the baseline sisal/PLA composite (17.52 kg CO_2_-eq) appears to have a higher environmental impact than aluminium (17.23 kg CO_2_-eq). However, when normalised using the material index to reflect structural performance requirements, the biocomposite’s lower density and higher relative compliance require 39.90 kg to match the bending stiffness of 28.20 kg of aluminium. Under the optimised Scenario A, the performance-based GWP of the sisal/PLA frame (391.8 kg CO_2_-eq) is 19.3% lower than that of the aluminium frame (485.9 kg CO_2_-eq), highlighting its environmental competitiveness [35].

Conversely, softwood and modified timber frames remain the most environmentally benign options, with a structural GWP of only 13.0 kg CO_2_-eq, owing to their low density, high natural stiffness (M = 4.31), and low-energy processing [35]. However, the optimised sisal/PLA composite shows potential as an alternative material, particularly where timber is structurally restricted due to durability issues, or where high moisture exposure limits timber’s service life [5,55]. Despite virgin PVC frames having an intermediate GWP, they pose severe ecotoxicity and end-of-life recycling challenges [56]. Thus, the optimised sisal/PLA biocomposite offers a bio-based, circular alternative that avoids the toxic chemicals and fossil-resource use associated with conventional plastics [12].

### Environmental, mechanical, and economic trade-offs

#### Toxicity burden-shifting mechanics and mitigation strategies.

A critical observation from the sensitivity analysis is the substantial increase in toxicity-related impacts under the optimisation scenarios. Specifically, while Scenario A reduces GWP by 43.97%, it increases Human Non-Carcinogenic Toxicity Potential (HNCTP) by 125.91%, Terrestrial Ecotoxicity Potential (TETP) by 10.16%, and Freshwater Ecotoxicity Potential (FETP) by 322.88%. The primary driver of this burden-shifting is the marine freight transport dataset. Container ships burn Heavy Fuel Oil (HFO), which contains high levels of sulfur, vanadium, nickel, and lead [53]. When air transport is replaced by marine transport, these toxic heavy-metal emissions are released directly into marine and terrestrial environments through engine combustion and leaching of copper-based antifouling paints from hulls.

Additionally, the upstream agricultural phase in Tanzania is a major contributor to these toxicity metrics. The extraction and processing of synthetic phosphate fertilisers used in sisal nurseries release cadmium, zinc, and lead, which accumulate in the soil and in water runoff during the fibre-washing phase. Shifting from a 28% to a 40% fibre loading in Scenario A increases the mass allocation of agricultural inputs per unit of finished composite, thereby propagating these toxicity burdens [51,57]. To mitigate toxicity burdens across the sisal/PLA biocomposite supply chain, several industrial strategies should be adopted. First, transitioning to low-sulphur marine fuels such as liquefied natural gas (LNG), marine gas oil (MGO), or biofuels can substantially reduce particulate, heavy-metal, and sulphur emissions during ocean transport [53]. Second, implementing organic agricultural management practices in Tanzanian sisal nurseries, including replacing synthetic phosphate fertilisers with organic compost, manure, and bio-based crop-protection alternatives, can directly minimise heavy-metal contamination in soil and runoff. Finally, at the manufacturing stage, replacing conventional sodium hydroxide (NaOH) chemical treatments with water-based thermal processing or bio-based enzymatic fibre-treatment methods can significantly reduce or eliminate toxic chemical effluents, thereby improving the overall environmental sustainability of the production system.

#### Mechanical–environmental performance trade-offs.

The sensitivity analysis demonstrates that reducing the PLA matrix content from 72 wt% to 60 wt% decreases Land Use and Fossil Resource Scarcity by 29.14% and 49.95%, respectively, primarily due to the reduced demand for biopolymer synthesis. While this reduction improves environmental performance, it also introduces important mechanical and durability trade-offs that may compromise the biocomposite’s structural integrity and long-term performance.

At higher fibre loadings (40 wt%, as represented in Scenario A), the viscosity of the molten polymer mixture increases significantly during processing, limiting the matrix’s ability to fully impregnate and wet the sisal fibre bundles. This incomplete wetting promotes fibre agglomeration and the formation of microstructural voids, which act as stress concentration points, weakening the overall composite structure [17]. Such defects can reduce load transfer efficiency between the matrix and reinforcement, ultimately diminishing tensile and flexural properties [58].

Beyond environmental considerations, increasing fibre loading introduces manufacturing challenges associated with fibre alignment, melt viscosity, resin impregnation, void formation, and dimensional consistency. These challenges have been widely recognised in continuous natural-fibre composite manufacturing and are particularly important during filament winding and additive manufacturing, where process control strongly influences fibre orientation, interfacial bonding, and final mechanical performance [59,60]. Consequently, industrial-scale production will require automated monitoring systems that can accommodate the inherent variability of natural fibres while ensuring consistent product quality [24]. This mismatch in moisture affinity facilitates greater water uptake, leading to fibre swelling, matrix micro-cracking, and interfacial degradation over time. These degradation mechanisms can significantly reduce tensile and flexural strength over the material’s service life, particularly in outdoor construction applications exposed to variable humidity and rainfall [59].

In offsetting the performance limitations of natural fibre-based composites, surface-chemical treatments using sodium hydroxide (NaOH) are often applied to remove hemicellulose, lignin, and other surface impurities, thereby improving fibre–matrix adhesion and reducing the risk of interfacial delamination [59]. However, such chemical treatments introduce additional environmental burdens, particularly toxicological emissions and wastewater treatment requirements. This creates a critical trade-off between achieving interfacial stability and increased environmental toxicity, highlighting the need for optimised fibre treatment strategies that balance structural and sustainability objectives [57].

### Limitation of the study

This study has several limitations. First, the assessment is restricted to a cradle-to-gate system boundary because the composite remains at prototype stage. Second, the manufacturing inventory was developed using pilot-scale production data, which may differ from future industrial-scale operations. Third, the study focused primarily on environmental performance and did not include comprehensive techno-economic or social sustainability assessments. These limitations should be addressed through future cradle-to-grave and industrial-scale investigations.

## Conclusions and recommendations

### Conclusions

This study presents a rigorous cradle-to-gate life cycle assessment of a transcontinental sisal fibre-reinforced polylactic acid (PLA) composite. Using the ReCiPe 2016 Midpoint (H) method, the environmental profile is dominated by a few critical hotspots, primarily intercontinental transport, composite manufacturing, and agricultural fibre extraction. Under baseline R&D pilot conditions, intercontinental air freight accounts for exactly 62.53% of total emissions, emphasising that early-stage product logistics impose heavy carbon penalties.

Transitioning to sea freight, adopting wind-based manufacturing grids, and reducing the PLA resin content in the matrix reduce the GWP by over 43.97%, making the biocomposite’s performance-based GWP structurally competitive with PVC and aluminium window frames. However, the analysis reveals a significant burden-shifting trade-off: these changes in logistics and composition increase human non-carcinogenic toxicity by 125.91% and freshwater ecotoxicity by 322.88%, likely driven by heavy fuel oil combustion in marine shipping and synthetic fertilisers used in agricultural cultivation. These findings demonstrate that carbon footprint reductions alone do not guarantee overall environmental sustainability, underscoring the necessity of using multi-indicator LCA frameworks for sustainable material selection.

Overall, the findings demonstrate that sustainable material selection should not rely exclusively on carbon footprint but instead integrate multiple environmental indicators, structural functionality, manufacturing feasibility, and economic considerations. Such multi-criteria decision-making frameworks are increasingly recognised as essential for evaluating emerging sustainable technologies because they minimise burden shifting while supporting robust industrial decision-making [30,60].

### Recommendations for future studies

The transition from laboratory-scale prototypes to commercial production requires simultaneous optimisation of environmental performance, manufacturing reliability, and economic viability. Industrial implementation should therefore prioritise maritime logistics, renewable electricity integration, automated filament winding, digital process monitoring, and optimisation of fibre-treatment technologies to ensure consistent product quality while maintaining environmental benefits [24,59]. These improvements should be evaluated alongside comprehensive techno-economic analyses before large-scale commercial deployment.

In that respect, a logical first step for future studies is to integrate the life cycle assessment (LCA) data from this work with a rigorous Techno-Economic Analysis (TEA) to evaluate the financial viability of the proposed optimisation measures. Specifically, the logistical shift rom from air to sea freight represents a vital trade-off; while it reduces GWP by 43.9% and offers substantial transport cost savings on a kg-km basis, it extends transit lead times to over 30 days. So, future studies must therefore model the financial impacts of increased inventory holding volumes and larger warehouse capacities against these operational savings.

Additionally, the processing parameters and mechanical trade-offs of the composite itself require further structural validation. For instance, the environmental benefits of increasing fibre loading to a 60:40 (PLA: sisal) ratio reduce land-use impacts by 29.1% and leverage cheaper raw sisal yarn relative to primary PLA resin, but it simultaneously introduces processing vulnerabilities. Future experimental work should focus on optimising filament winding tensioners and manufacturing windows to prevent fibre clumping, void formation, and mechanical deterioration. Similarly, while a water-based thermal treatment is proposed as it eliminates the chemical toxicity of NaOH, future studies must evaluate whether its high thermal energy demand and extended processing times outweigh its ecological benefits at scale.

Beyond localised processing adjustments, long-term commercialisation strategies can be significantly advanced by investigating the integration of Industry 5.0 principles to safeguard against transcontinental supply chain disruptions, biological feedstock variability, and long transit delays [24]. Moreover, digitalising the sisal supply chain through real-time sensors could improve resource efficiency and traceability. For example, soil moisture sensors in Tanzanian plantations and temperature and humidity sensors in maritime cargo containers can optimise fibre production, monitor transport conditions, and reduce material losses. In addition, machine learning-based process control during filament winding and compression moulding could automatically adjust winding tension and cooling rates to accommodate natural fibre variability, thereby improving composite quality and manufacturing consistency [24,31].

Future research should investigate the use of nanomaterial reinforcements, including graphene oxide (GO), reduced graphene oxide (rGO), and graphene nanoplatelets, to enhance interfacial bonding, mechanical performance, moisture resistance, and multifunctional self-sensing capabilities of sisal/PLA composites [43,61]. Furthermore, extending the assessment from a cradle-to-gate to a cradle-to-grave framework would enable a more comprehensive evaluation of use-phase maintenance and end-of-life scenarios, including protective coatings, industrial composting, mechanical recycling, and comparisons with established recycling facilities.

## Use of Generative AI

During the preparation of this work, Grammarly was used solely as a writing-assistance tool to improve grammar, spelling, punctuation, and clarity of language. No generative AI tools were used to generate, analyse, interpret, or revise the scientific content of the manuscript. All language suggestions generated by Grammarly were carefully reviewed, verified, and accepted or rejected by the authors before being incorporated into the manuscript. The authors take full responsibility for the accuracy, integrity, and originality of the submitted work.

## Supporting information

S1 File Monte Carlo simulation data and calculations for the baseline model using 10,000 iterations. (XLSX)
